# Supplementary material for: Mechanisms governing the pioneering and redistribution capabilities of the non-classical pioneer PU.1
Source: Nat Commun. 2020 Jan 21;11:402. doi: 10.1038/s41467-019-13960-2 (PMC6972792; doi:10.1038/s41467-019-13960-2)
Supplement: Supplementary file 7 — Source data [file 41467_2019_13960_MOESM7_ESM.zip › Source_Data/Figure6/Figure6B_MotifScanOutput/ETS1_mutPU.1/homerResults/motif39.similar.html]

motif39

## Information for motif39

C
A
G
T
T
C
A
G
T
G
A
C
T
C
A
G
A
T
G
C
G
T
C
A
C
A
G
T
A
T
C
G
A
G
T
C
C
A
T
G
A
G
T
C
  
Reverse Opposite:  

T
C
A
G
G
T
A
C
T
C
A
G
A
T
G
C
G
T
C
A
C
A
G
T
T
A
C
G
A
G
T
C
A
C
T
G
A
G
T
C
G
T
C
A
  

|  |  |
| --- | --- |
| p-value: | 1e-38 |
| log p-value: | -8.889e+01 |
| Information Content per bp: | 1.687 |
| Number of Target Sequences with motif | 438.0 |
| Percentage of Target Sequences with motif | 3.25% |
| Number of Background Sequences with motif | 474.9 |
| Percentage of Background Sequences with motif | 1.35% |
| Average Position of motif in Targets | 101.8 +/- 55.6bp |
| Average Position of motif in Background | 94.7 +/- 70.6bp |
| Strand Bias (log2 ratio + to - strand density) | -0.0 |
| Multiplicity (# of sites on avg that occur together) | 1.16 |
| Motif File: | file (matrix) reverse opposite |

### Similar de novo motifs found

|  |  |  |  |  |  |  |  |
| --- | --- | --- | --- | --- | --- | --- | --- |
| Rank | Match Score | Redundant Motif | P-value | log P-value | % of Targets | % of Background | Motif file |
| 1 | 0.991 | T A G C A C G T T C A G T G A C T C A G A G T C T G C A C A G T A T C G G A T C C A T G A G T C | 1e-36 | -83.476257 | 3.00% | 1.24% | motif file (matrix) |
| 2 | 0.955 | C G A T T C A G T G A C T C A G G T A C G T C A C A G T A C T G A G T C C A T G | 1e-35 | -80.994842 | 2.62% | 1.02% | motif file (matrix) |
| 3 | 0.982 | A T G C T C G A A T G C A G C T T C A G T G A C C T A G A G T C T G C A A C G T A C T G A G T C A C T G A G T C | 1e-34 | -78.495714 | 2.90% | 1.21% | motif file (matrix) |
| 4 | 0.984 | A G C T T C A G T G A C T C A G A G T C T G C A A C G T A C T G A G T C A C T G G A T C T C G A T A C G | 1e-32 | -74.234402 | 2.88% | 1.24% | motif file (matrix) |
| 5 | 0.879 | C T A G T G A C T C A G A T G C G C T A C A G T T A C G A G T C C A T G | 1e-27 | -62.577208 | 3.58% | 1.85% | motif file (matrix) |
| 6 | 0.753 | A T G C C T A G A T G C G C A T G C T A C A T G T G A C C T A G | 1e-13 | -31.022386 | 1.50% | 0.73% | motif file (matrix) |
